# Feasibility and accuracy of real-time 3D-holographic graft length measurements

**DOI:** 10.1093/ehjdh/ztad071

**Published:** 2023-11-14

**Authors:** Tsung-Ying Tsai, Shigetaka Kageyama, XingQiang He, Giulio Pompilio, Daniele Andreini, Gianluca Pontone, Mark La Meir, Johan De Mey, Kaoru Tanaka, Torsten Doenst, John Puskas, Ulf Teichgräber, Ulrich Schneider, Himanshu Gupta, Jonathon Leipsic, Scot Garg, Pruthvi C. Revaiah, Maciej Stanuch, Andrzej Skalski, Yoshinobu Onuma, Patrick W Serruys

**Affiliations:** Cardiovascular center, Taichung Veterans General Hospital, Taichung, Taiwan; Corrib Research Centre for Advanced Imaging and Core Laboratory, University of Galway, University Road, H91 TK33, Galway, Ireland; Corrib Research Centre for Advanced Imaging and Core Laboratory, University of Galway, University Road, H91 TK33, Galway, Ireland; Corrib Research Centre for Advanced Imaging and Core Laboratory, University of Galway, University Road, H91 TK33, Galway, Ireland; Department of Cardiovascular Surgery, Centro Cardiologico Monzino IRCCS, Milan, Italy; Department of Biomedical, Surgical and Dental Sciences, University of Milan, Milano, Italy; Department of Biomedical, Surgical and Dental Sciences, University of Milan, Milano, Italy; Division of Cardiology and Cardiac Imaging, IRCCS Galeazzi Sant’Ambrogio, Milan, Italy; Department of Biomedical and Clinical Sciences, University of Milan, Milano, Italy; Department of Biomedical, Surgical and Dental Sciences, University of Milan, Milano, Italy; Department of Perioperative Cardiology and Cardiovascular Imaging, Centro Cardiologico Monzino IRCCS, Milan, Italy; Department of Cardiac Surgery, Universitair Ziekenhuis Brussel, VUB, Brussels, Belgium; Department of Radiology, Universitair Ziekenhuis Brussel (UZ Brussel), Belgium; Department of Radiology, University Hospital Brussels, Brussels, Belgium; Department of Cardiothoracic Surgery, University Hospital Jena, Jena, Germany; Department of Cardiovascular Surgery, Mount Sinai Morningside, NewYork, USA; Department of Radiology, University Hospital Jena, Jena, Germany; Department of Cardiothoracic Surgery, University Hospital Jena, Jena, Germany; Department of Cardiology and Radiology, The Valley Hospital, Ridgewood, NJ, USA; Centre for Cardiovascular Innovation, St.Paul’s Hospital, University of British Columbia, Vancouver, British Columbia, Canada; Department of Cardiology, Royal Blackburn Hospital, Blackburn, United Kingdom; Corrib Research Centre for Advanced Imaging and Core Laboratory, University of Galway, University Road, H91 TK33, Galway, Ireland; MedApp S.A., Kraków, Poland; Department of Measurements and Electronics, AGH University of Krakow, Kraków, Poland; MedApp S.A., Kraków, Poland; Department of Measurements and Electronics, AGH University of Krakow, Kraków, Poland; Corrib Research Centre for Advanced Imaging and Core Laboratory, University of Galway, University Road, H91 TK33, Galway, Ireland; Corrib Research Centre for Advanced Imaging and Core Laboratory, University of Galway, University Road, H91 TK33, Galway, Ireland

**Keywords:** Augmented reality, Mixed reality, Coronary artery bypass graft, Computed tomography, Hologram

## Abstract

**Aims:**

Mixed reality (MR) holograms can display high-definition images while preserving the user’s situational awareness. New MR software can measure 3D objects with gestures and voice commands; however, these measurements have not been validated. We aimed to assess the feasibility and accuracy of using 3D holograms for measuring the length of coronary artery bypass grafts.

**Methods and results:**

An independent core lab analyzed follow-up computer tomography coronary angiograms performed 30 days after coronary artery bypass grafting in 30 consecutive cases enrolled in the FASTTRACK CABG trial. Two analysts, blinded to clinical information, performed holographic reconstruction and measurements using the CarnaLife Holo software (Medapp, Krakow, Poland). Inter-observer agreement was assessed in the first 20 cases. Another analyst performed the validation measurements using the CardIQ W8 CT system (GE Healthcare, Milwaukee, Wisconsin). Seventy grafts (30 left internal mammary artery grafts, 31 saphenous vein grafts, and 9 right internal mammary artery grafts) were measured. Holographic measurements were feasible in 97.1% of grafts and took 3 minutes 36 s ± 50.74 s per case. There was an excellent inter-observer agreement [interclass correlation coefficient (ICC) 0.99 (0.97–0.99)]. There was no significant difference between the total graft length on hologram and CT [187.5 mm (157.7–211.4) vs. 183.1 mm (156.8–206.1), *P* = 0.50], respectively. Hologram and CT measurements are highly correlated (r = 0.97, *P* < 0.001) with an excellent agreement [ICC 0.98 (0.97–0.99)].

**Conclusion:**

Real-time holographic measurements are feasible, quick, and accurate even for tortuous bypass grafts. This new methodology can empower clinicians to visualize and measure 3D images by themselves and may provide insights for procedural strategy.

## Structured Graphical Abstract

**Structured Graphical Abstract:**
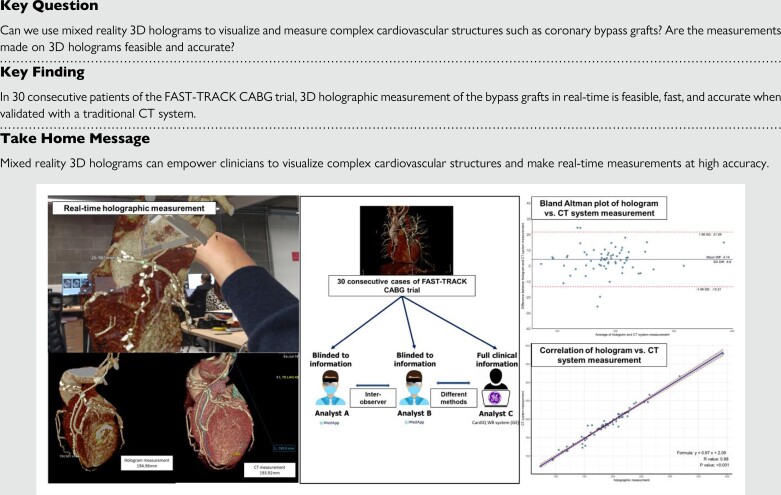

## Introduction

Three-dimensional (3D) imaging modalities have been indispensable in driving the rapid development of cardiovascular interventions in the past decade,^1^ however, they are limited by the 2-dimensional display of computer screens. Numerous extended reality technologies have, therefore, been developed to bring 3D images into the physical world as 3D holograms. Among them, mixed reality (MR) technology can simultaneously display high-definition 3D holograms while preserving the user’s situational awareness, making it the ideal tool for pre-procedural planning and intra-procedural visualization.^2^ MR technologies have been implemented in many clinical scenarios, such as surgery for congenital heart disease or structural heart intervention.^3,4^ With high-precision motion capture technology onboard state-of-the-art MR headsets, users of MR can manipulate 3D images with gestures and voice commands without breaking sterility, including making measurements of real objects or 3D holograms. The measurement of real-world objects with MR headsets has been tested for medical use in small experiments.^5^ However, measurement on 3D-rendered holograms, which can be expanded/shrunk, rotated, and cut by the user, is a whole different difficulty level. Thus, an additional study is warranted to assess whether mixed-reality measurement on 3D holograms is a feasible and accurate modality.

Coronary computed tomographic angiography (CCTA) is a non-invasive and highly sensitive modality for diagnosing coronary artery disease (CAD) and is ideal for the assessment of graft patency after coronary artery bypass graft (CABG) surgery.^6^ Graft tension, overstretching, kinking, or redundancy are important mechanisms for early graft failure.^7^ Post-CABG CCTA offers a unique window to assess graft length and trajectory; however, these measurements are time-consuming and technically challenging and, thus, not routinely reported by radiologists in clinical practice. However, for surgeons, this knowledge may help improve surgical planning in the future. In this study, we aim to assess the feasibility and accuracy of using CT-derived 3D holograms for measuring the length of coronary artery bypass grafts.

## Methods

We prospectively analyzed the 30-day post-CABG follow-up CCTA of 30 consecutive cases in the FAST-TRACK CABG trial, which is an investigator-initiated single-arm, multicenter, prospective study aiming to prove the feasibility and safety of planning surgical revascularization solely based on CCTA, without knowledge of the anatomy defined by invasive coronary angiography.^8^ All patients in this study received post-operative CCTA scans at 30-day follow-up using the Revolution computed tomography (CT) scanners (GE Healthcare, Milwaukee, WI, USA), which have a nominal spatial resolution of 230μm along the X–Y planes, a rotational speed of 0.28 s, and a Z-plane coverage of 16 cm enabling imaging of the heart in one heartbeat. All patients received nitrates before CCTA acquisition and beta-blockers in cases of heart rates ≥65 bpm. Image quality was controlled by expert reviewers using the five-point Likert scale at patient and segment levels.

**Figure 1:**
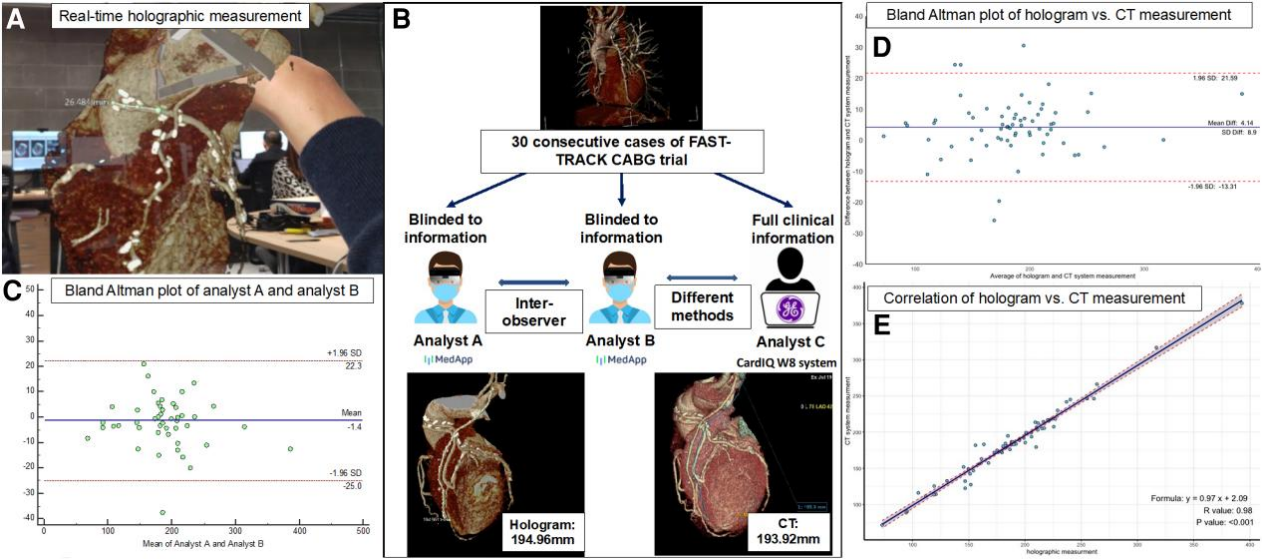
Study workflow and results. Panel A shows an example of real-time measurements on holographic reconstructions with hand gestures and voice commands. Panel B shows the study workflow. Thirty consecutive FAST TRACK CABG trial cases were analyzed using the traditional CT workstation or 3D holograms. An example was provided in the lower panel. Panel C shows the Bland-Altman plot of the holographic graft length measurement in the first 20 cases by two analysts. Panel D shows the Bland-Altman plot of the graft length measurement by hologram vs. CT system. Panel E shows the correlation between the holographic and traditional CT system measurements.

The CCTA images were transferred and analyzed by an independent core lab (Corrib, Galway, Ireland), where they were processed using the CarnaLife Holo software (Medapp, Krakow, Poland) for hologram reconstruction and the CardIQ W8 CT system (GE Healthcare, Milwaukee, Wisconsin) for validation analysis. The 3D holograms were pre-processed by removing the chest wall, rib cage, and posterior mediastinal structures with the built-in scissor tool, which takes approximately 3 min per case. Two analysts (TT, XH) blinded to clinical information used the CarnaLife Holo software and a HoloLens 2 headset (Microsoft, Redmond, Washington, USA) to perform real-time holographic graft length analysis with voice command and hand gestures (Figure  *1A*). The third analyst (S.K.), with full access to clinical information, performed validation measurements on the CardIQ W8 CT system following the automatically generated vessel centrelines with manual adjustments (Figure  *1B*). Graft lengths were measured from the subclavian artery [for internal thoracic arteries (IMA)] or the aorta [for saphenous vein graft (SVG)] to the last anastomosis to the coronary arteries. The inter-observer agreement was assessed in the first 20 cases to evaluate the consistency of holographic analysis.

Quantitative variables are reported as mean ± standard deviation (SD) or median and interquartile range (interquartile range, 25–75%) according to distribution. Categorical variables are expressed as numeric values and percentages. The comparison between the CT and holographic measurement was done using the non-parametric Mann–Whitney *U* test or paired-sample *t*-test when appropriate. The Pearson correlation, intra-class correlation coefficients (ICC), and Bland–Altman method were used to quantify the correlation between paired graft length measurement with hologram and CT system. A two-sided *P*-value < 0.05 was considered to be statistically significant. All data were processed using SPSS version 27.0 (IBM Inc, Armonk, NY, USA) and R 4.1.1 (The R Foundation for Statistical Computing, Vienna, Austria).

## Results

A total of 70 grafts (30 left IMA grafts, 31 SVG, and 9 right IMA graft) were analyzed (*Table 1*). The feasibility of graft length measurement with CarnaLife Holo and CardIQ W8 was both 97.1% (all grafts were analyzable except for an SVG, and a right IMA graft occluded on 30-day follow-up CCTA). On average, the holographic measurement took 3 min and 36 s ± 50.7 s per case compared to around 20 min per case on the CT system. There was excellent inter-observer agreement in the length measurement of the 47 grafts of the first 20 patients using hologram software, with median graft lengths measured by analysts A and B of 187.5 mm (170.0–209.6) and 187.5 mm (157.7–211.4), respectively [mean difference 1.4 ± 9.1 mm, 95% lower limit of agreement (LLA) −25 mm and upper limit of agreement (ULA) 22.3 mm] with an ICC of 0.99 (0.98–0.99, Figure  *1C*). There was no significant difference between the graft length measured with the hologram or CT system 187.5 mm (157.7–211.4) vs. 183.1 mm (156.8–206.1), *P* = 0.50. There was also no significant difference between modalities in the length measurement across different graft types (left IMA, SVG, right IMA), as shown in *Table 1*. The Bland–Altman plot showed that the mean difference between the CT system and holographic graft length measurement was 4.14 ± 8.9 mm, (95% LLA −13.31 and ULA 21.59, Figure  *1D*). The measurements on hologram and CT were highly correlated (r = 0.97, *P* < 0.001) with an excellent agreement [ICC 0.98(0.97–0.99), Figure  *1E*].

**Table 1:** Graft length measurement with hologram and CT

	Hologram	CT system	*P*-value^a^	ICC
Overall	187.5 (157.7–211.4)	183.1 (156.8–206.1)	0.50	0.98 (0.97–0.99)
LIMA (*n* = 30)	186.1 ± 21.5	181.3 ± 16.6	0.42	0.92 (0.85–0.96)
GSV (*n* = 31)	167.5 ± 52.3	164.9 ± 54.1	0.80	0.98 (0.95–0.99)
RIMA (*n* = 9)	255.2 ± 67.2	245.3 ± 67.0	0.38	0.99 (0.94–1.00)

ICC = intra-class correlation coefficients, LIMA = left internal mammary artery, GSV = great saphenous vein graft, RIMA = right internal mammary artery.

^a^ *P*-value of comparison between the CT and holographic measurement using the non-parametric Mann–Whitney *U* test or paired-sample *t*-test.

## Discussion

In this small prospective feasibility study, we demonstrated that holographic measurements, enabled using MR technology, offer an accurate and efficient option for evaluating complex cardiovascular structures in real time. This enables operators to fully leverage the power of advanced imaging and reduce the time spent at the imaging workstation after a short training session to familiarize themselves with the software. MR measurements offer the opportunity for clinicians to make custom measurements that are not routinely provided; the length of bypass grafts is a perfect example of such clinician-initiated measurements. The ability to freely measure the graft length would allow the surgeons to appreciate the ‘in vivo’ length of the graft implanted and to appreciate whether the graft material is in excess or insufficient. This may help them assess the effect of graft material treatment, for instance, skeletonization of arterial grafts or different vasodilator treatments, on graft length. With the ability to intuitively make measurements on 3D holograms, surgeons can potentially plan the precise length of arterial and venous grafts that need to be harvested for each CABG operation, reducing unnecessary tissue loss and potentially the risk of early graft failure.^7,9^ In addition, MR tools can potentially grant clinicians the mobility to leave computer screens and visit the bedside, whilst the intuitive images can also help engage patients in interactive education and discussion sessions.

MR technology also offers the unique ability to bring 3D images, including echocardiography, CT, and magnetic resonance image to the operating table for the operator to examine at their fingertips. This may prove essential in complex procedures and operations that require detailed planning and measurements on images, for instance, planning the size of surgical implants. In addition, should the need for additional measurements arise, these can be performed immediately *ad-hoc* and without breaking sterility. The next iteration of MR would be even more powerful with the fusion of multiple imaging modalities, including fluoroscopy, and echocardiography.^10^

Our study marks the first step in the evolution of 3D holograms from a pure visualization tool to an interactive imaging modality for real-time reference. Although we showed excellent agreement between holographic graft length measurements and their actual length, further study with other types of measurement, such as quantified tortuosity, area, or volume, and in other clinical scenarios, including structural heart disease, or percutaneous coronary intervention, should be performed to expand the generalizability of hologram measurements. 3D holograms measurements on other modalities, such as echocardiography and intravascular imaging, are potential applications of this technology in the future.

## Conclusion

Real-time holographic measurements are feasible, quick, and accurate even for tortuous coronary bypass grafts. This new methodology enables surgeons to visualize and measure the results of their work by themselves and may provide insights for procedural strategy. Eventually, these 3D holograms may empower surgeons to plan the precise length of arterial and venous grafts to be harvested for every individual case.

**Institutional Review Board (IRB) Approval:** IRB approval was obtained at each individual participating centre.

## Informed consent statement

Written consent was obtained for all patients.

## Data Availability

Data are subject to an embargo and might be shared in the future after the publication of the FAST TRACK CABG main result paper.
